# P-2110. Evaluating Different Screening Methods to Assess the Prevalence of Gut Colonization with Multidrug-Resistant Gram-Negative *Bacilli* in Hospitalized Patients

**DOI:** 10.1093/ofid/ofae631.2266

**Published:** 2025-01-29

**Authors:** Rebecca A Tenner, Maria Paz Riquelme, Johanna Acevedo, Lea Paz Maureira Quezada, Maite Loreto Valentina Gonzalez Soto, Jose R W Martínez, Lorena Diaz, Anne S Peters, Rafael Araos, Jose M Munita

**Affiliations:** Tufts University (Boston, MA, USA) | Facultad de Medicina - Clínica Alemana Universidad del Desarrollo (Santiago, Chile), New York, New York; Universidad del Desarrollo, Santiago, Region Metropolitana, Chile; Universidad del Desarrollo, Santiago, Region Metropolitana, Chile; Instituto de Ciencias e Innovación en Medicina, Universidad del Desarrollo, Santiago de Chile, Santiago, Region Metropolitana, Chile; Universidad del Desarrollo, Santiago, Region Metropolitana, Chile; Genomics & Resistant Microbes (GeRM), Instituto de Ciencias e Innovación en Medicina, Facultad de Medicina Clínica Alemana, Universidad del Desarrollo, Chile; Millennium Initiative for Collaborative Research on Bacterial Resistance (MICROB-R), Santiago, Region Metropolitana, Chile; Univerdidad del Desarrollo, Santiago, Region Metropolitana, Chile; Universidad del Desarrollo, Santiago, Region Metropolitana, Chile; Universidad del Desarrollo, Santiago, Region Metropolitana, Chile; Facultad de Medicina Clinica Alemana - Universidad del Desarrollo, Santiago, Region Metropolitana, Chile

## Abstract

**Background:**

Accurate estimates of gut colonization with drug-resistant gram-negative bacilli (GNB) are key to combat antimicrobial resistance. Data on best practices to assess colonization is scarce, limiting the comparability and feasibility of screening efforts. Building upon ongoing projects studying gut colonization with carbapenem-resistant (CR), carbapenemase-producing (CP-CR) and extended-spectrum beta-lactamase-producing (ESBL) GNB in Chile, we compared 3 different screening strategies and assessed the impact of time between swab collection to plating.

Prevalence of Gut Colonization with Multidrug-Resistant Gram-Negative Bacilli Detected with Different Screening Methods

**Figure:**
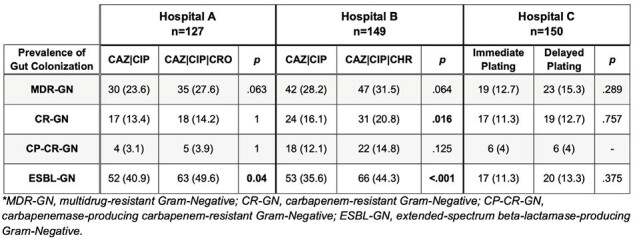

**Methods:**

Rectal swabs were collected for a gut colonization point-prevalence study in 3 Chilean hospitals (n=127, 149, 150). All swabs were plated within 2h of collection onto separate MacConkey agars with CAZ 2µg/mL and CIP 2µg/mL (CAZ|CIP). In hospital A samples were also plated on MacConkey + ceftriaxone 2µg/mL (CAZ|CIP|CRO). In hospital B swabs were also plated onto CHROMagar ESBL^TM^ (CAZ|CIP|CHR). Swabs from Hospital C were plated on CAZ|CIP within 2h(immediate) and were then re-streaked after 48h at room temperature (delayed plating). After 24h of incubation at 37°C, all isolated morphotypes were characterized. Prevalence of gut colonization between CAZ|CIP, CAZ|CIP|CRO and CAZ|CIP|CHR were compared, as were plating times for hospital C.

**Results:**

Results are summarized in Table 1. Prevalence of gut colonization in hospital A using CAZ|CIP vs. CAZ|CIP|CRO was similar for MDR-GNB (24% vs 28%), CR-GNB (13% vs 14%), and CP-CR-GNB (3% vs 4%). The addition of CRO detected a higher prevalence of ESBL-GNB compared to CAZ|CIP alone (50% vs 41%; *p*=0.04). In hospital B, CAZ|CIP|CHR detected a higher prevalence of colonization with CR-GNB (21% vs 16%, *p*=0.016) and ESBL-GNB (44% vs 36%, *p* < 0.001), but similar results for MDR-GNB and CP-CR-GNB as compared to CAZ|CIP. The prevalence of gut colonization did not vary between immediate vs. delayed plating (Hospital C).

**Conclusion:**

The addition of a CRO or CHR agar increased the detection of gut colonization with ESBL-GNB compared to a CAZ|CIP protocol. CHR also provided a higher yield of CR-GNB. However, observed CP-CR-GNB prevalence was similar among all 3 conditions. Finally, a delayed plating strategy (48h) did not impact colonization detection as compared to immediate plating (2h).

**Disclosures:**

Jose M. Munita, MD, MSD: Grant/Research Support|Pfizer: Grant/Research Support

